# CPR during COVID-19: Use of Expert-driven Rapid Cycle Deliberate Practice to Implement PALS Guidelines

**DOI:** 10.1097/pq9.0000000000000374

**Published:** 2020-12-28

**Authors:** Blake E. Nichols, Ali B.V. McMichael, A. Paige Davis Volk, Priya Bhaskar, Cindy Darnell Bowens

**Affiliations:** From the Department of Pediatrics, University of Texas Southwestern Medical Center, Dallas, Tex.

## Abstract

The American Heart Association (AHA) and other national institutions have endorsed modifications to resuscitation guidelines given the risk of healthcare workers’ (HCWs) exposure to COVID-19. Institutional implementation of the COVID-19-focused guidelines requires both proof of feasibility and education of HCW. Pediatric critical care medical directors at The University of Texas Southwestern/Children’s Health System of Texas (UTSW/CHST) created a guideline for the resuscitation of COVID-19 patients. The simulation team used in situ simulation to demonstrate guideline feasibility and to create educational materials.

**Methods:**

A UTSW/CHST guideline incorporated COVID-19-focused AHA and other national organizational recommendations to fit the institutional needs. A high-fidelity in situ simulation helped test the feasibility and optimize the UTSW/CHST guideline. We developed a novel form of rapid cycle deliberate practice (RCDP), expert-driven RCDP, in which all simulation participants are experts, to debrief the simulation.

**Results:**

In situ simulation with expert-driven RCDP demonstrated guideline feasibility in the resuscitation of a COVID-19 patient while balancing the protection of HCW. Expert-driven RCDP allowed for real-time alterations to the guideline during the simulation event. Video recording and dissemination of the simulation allowed for the education of over 300 staff on the new recommendations.

**Conclusions:**

High-fidelity in situ simulation with expert-driven RCDP created a rapid consensus among expert critical care providers to develop the UTSW/CHST guideline and quickly adopt the new AHA recommendations. This debriefing method helped minimize the risk of HCW exposure by minimizing the number of required participants and time for simulation. We recommend using this distinctive, expert-driven RCDP debriefing method for expeditious testing of COVID-19-focused processes at other institutions.

**Video Abstract available at:** [link forthcoming]

## INTRODUCTION

Pediatric intensive care units (PICUs) worldwide struggle to balance accepted best practices for pediatric resuscitation with the protection of healthcare workers (HCWs) during the COVID-19 pandemic. As many as 12%–19% of COVID-19 positive adult patients require hospital admission, 3%–6% require intensive care.^1,2^ As of May 30, 2020, only 5.2% of COVID-19 positive cases are children below 20 years of age. Over 2,000 afflicted children in the United States have been admitted to hospitals, with 357 to PICUs, which demonstrates that COVID-19 will likely cause a significant disease burden for children.^3–5^ Intubation and cardiopulmonary resuscitation (CPR) are among the highest risk procedures known to spread the COVID-19 virus through aerosolization.^6,7^ Recently, the Resuscitation Adult and Pediatric Task Forces of the American Heart Association (AHA) and the Society of Critical Care Medicine (SCCM) published guidelines for a unified approach to resuscitation practices during the COVID-19 pandemic.^8,9^ The pediatric critical care medical directors at the University of Texas Southwestern Medical Center/Children’s Health System of Texas (UTSW/CHST) created a guideline based on the AHA and SCCM recommendations for intubation and CPR of a pediatric COVID-19 positive patient or person under investigation (PUI).

The guideline includes modifications to standard intubation and resuscitation practices, which require re-education of the multiprofessional critical care team. Given the time-sensitive nature with which this guideline required implementation, we created multiprofessional in situ simulations of intubation and resuscitation of a COVID-19 patient or PUI. In situ simulation is used successfully in many medical disciplines for team-based crisis resource management education and high-risk procedural education to improve competence and performance in the learner’s work environment.^10,11^ Additionally, the faculty simulation team used rapid cycle deliberate practice (RCDP) debriefing, an established method that uses direct, immediate feedback to achieve near-perfect practice, to test the implementation of the guideline.^12^

The study’s primary goal is to provide clear guidance to a multiprofessional treatment team at CHST on optimizing care of pediatric COVID-19 patients or PUIs during respiratory failure or cardiopulmonary arrest while balancing the protection of HCW. The team provided the following at our institution: (1) an AHA-based COVID-19 resuscitation guideline; (2) a PICU in situ simulation video of intubation and CPR; (3) a written scenario with simulation instructions; and (4) printable PICU HCW educational materials including appropriate team member positioning during resuscitation and proper donning and doffing of personal protective equipment (PPE). In situ simulation of COVID-19-targeted intubation and resuscitation guidelines is critical for safe resuscitation at our large, pediatric quaternary care institution given the pandemic time constraints, the potential for harm to HCW, and the changes to standard care outlined by the AHA and SCCM recommendations.

## METHODS

### Guideline Development Process

The UTSW/CHST guideline-writing committee comprised 3 pediatric critical care medical directors, 1 from the main campus PICU, 1 from the cardiac ICU, and 1 from the satellite campus PICU, created institutional guidelines for the management of COVID-19 patients or PUIs by employing recently released recommendations from the AHA, Surviving Sepsis Campaign, State of Texas, CDC, and teleconferencing with local experts.^8,9,13–18^ The proposed COVID-19 patient resuscitation institutional guideline has several new elements compared with standard resuscitation practice. The new elements focused on the limitation of equipment, personnel, and environment to minimize exposure of HCW. Equipment limitation involves keeping code carts, airway carts, and drug boxes outside of the patient room. Personnel limitation involves excluding trainees, including fellow physicians, from resuscitation of COVID-19 patients or PUIs. Environmental limitation involves using negative pressure rooms with an anteroom as the patient location. The guideline-writing committee presented the first iteration of the guideline to the UTSW/CHST critical care division during a COVID-19-focused meeting. During the guideline presentation, the committee received feedback on the guideline, highlighting the need for an in situ simulation-based assessment of the guideline’s feasibility and implementation.

### Simulation Development Process

**Fig. 1.**
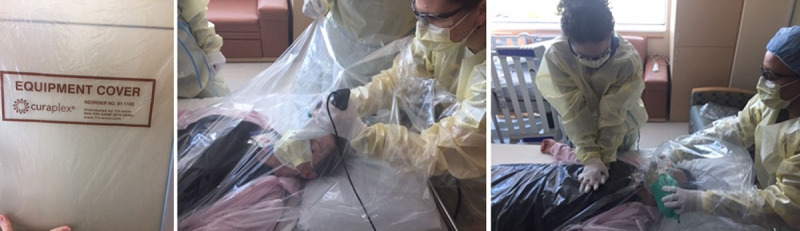
Feasibility of clear plastic drape. The clear plastic drape covers the patient from head-to-toe and may prevent HCW exposure to bodily fluids and droplets. The simulation team placed the drape over an embedded participant to simulate feasibility for intubation and cardiopulmonary resuscitation. The drape can be tucked under the patient to prevent slipping during cardiopulmonary resuscitation.

The simulation team developed a series of simulations to test guideline implementation of equipment use, resuscitation team dynamics, and environmental factors. First, the simulation team used an embedded participant to assess the use of a clear plastic Curaplex drape (Tri-anim Health Services, Sarnova Inc., Dublin, Ohio), which covers the patient during intubation and resuscitation, to minimize the spread of aerosolized viral particles and infected bodily fluids (Fig. 1). Second, the team created 2 high-fidelity simulation scenarios to test the feasibility and implementation of the new guideline. The first scenario required intubation for acute hypoxemic respiratory failure, and the second scenario required CPR of a COVID-19 patient.

The team developed and performed a high-fidelity in situ simulation in the PICU at a CHST hospital in a standard negative pressure room with anteroom to ensure environmental accuracy. The scenario utilized full PPE to increase the environmental fidelity of the simulation. Acknowledging the need to conserve PPE during the COVID-19 pandemic, all participants used clean, repurposed N95 masks, reusable goggles for eye protection rather than disposable face shields, and they did not double glove for intubation and resuscitation as is called for in some guidelines.

Expert participants in the simulation scenarios included pediatric critical care nurses, respiratory therapists, and fellow and attending physicians. The team recorded the scenario for dissemination to, and education of, approximately 300 critical care staff. All participants were aware of the scenario before the simulation, and all consented to photo and video recording use, following the standard UTSW talent release process. The simulation team debriefed using a modified version of RCDP, here referred to as expert-driven RCDP. As opposed to traditional RCDP, where participants in the simulation are learners, and the educator provides direct feedback, all participants in the room are considered experts. Any participant could call a time-out to stop, debrief, and adjust the scenario.^12^ The scenario resumed after clarification from each debrief. One of the guideline-writing committee members attended the simulation event to assist with the guideline’s adjustment and clarification.

### Testing of Guideline for Intubation of a COVID-19 Patient or PUI

The first high-fidelity simulation involved a pediatric patient with acute hypoxemic respiratory failure who failed noninvasive support and required intubation. The guideline dictated that only an attending pediatric critical care physician or pediatric anesthesiologist should intubate a patient to minimize attempts at securing the airway. Per the first guideline iteration, the simulation was initially designed to use 1 bedside nurse. Additional personnel included 1 respiratory therapist and 1 intubating physician during the intubation process. Limitations to personnel, equipment, and the environment were tested during the simulation. Table 1 shows the intubation checklist from the guideline.

**Table 1.** Intubation Checklist

∘ Identify intubating team: primary operator (MD), RT, 2 RNs
∘ Consider anesthesia as primary operator
∘ Prepare intubation equipment outside room including
∘ Video laryngoscopy complete with appropriate blade size
∘ ETT, stylet, clamp, syringe, HEPA filter
∘ ETCO2 monitor
∘ Oral airway
∘ Flow-inflating bag
∘ Curaplex clear plastic drape
∘ Two working peripheral IVs
∘ Code medications prepared and vasoactives at bedside if hemodynamically unstable
∘ Perform time out before intubation
∘ Induction medications at the bedside
∘ Strongly recommend RSI
∘ During intubation
∘ Two-hand seal on mask; avoid bagging
∘ Inflate ETT cuff after placement of ETT into airway and before bagging
∘ Clamp ETT before transition to mechanical ventilator (with HEPA filter in line)

ETCO2, end tidal carbon dioxide; ETT, endotracheal tube; IV, intravenous line; MD, medical doctor; RN, registered nurse; RT, respiratory therapist; RSI, rapid sequence intubation.

### Testing of Guideline for CPR of a COVID-19 Patient or PUI

The second high-fidelity simulation involved CPR after an acute respiratory arrest of the intubated COVID-19 patient. The first guideline iteration outlined that as few as 6 participants should perform standard resuscitation inside the patient room: 1 leader, 1 bedside nurse, 1 recorder, 1 respiratory therapist, and 2 team members for chest compressions. Additional roles might include an anesthesiologist to intubate during resuscitation and an additional attending or fellow physician to place a central line. Limitations to personnel, equipment, and the environment were tested during the simulation.

### Modification and Dissemination of Guideline

The committee modified the UTSW/CHST guideline during the simulation using expert-driven RCDP. Following the simulation, the recording was edited using iMovie (version 10.1.14) and disseminated to the multiprofessional critical care team members. Subtitles provided clarity for portions of the guideline for intubation and CPR of a patient with COVID-19 or PUI. The video was disseminated to critical care staff as required education via their well-established online educational platform.

## RESULTS

First, the simulation team found the Curaplex equipment cover to be superior to the plexiglass box as a protective barrier for HCW. The drape’s advantages over the plexiglass box include that it covers the entire patient, controls droplet and bodily fluid spread, allows for constant visualization of the patient, can be secured to the bed without tearing, and is flexible, which allows for manipulation of the patient through the drape. The drape is also portable, making it feasible for use by a code or rapid response team. Several disadvantages were observed with the drape, including use for covering patients during resuscitation as it can slip unless well secured. Additionally, it must be precut by staff and ready for use. Although the plexiglass box is a more stable physical and transparent barrier that can be secured, it is less available in our institution and less portable. It decreases the intubating physician’s mobility and resuscitation team members’ access to the patient more than the drape. Taken together, we considered the advantages of the drape to outweigh those of the plexiglass box.

**Fig. 2.**
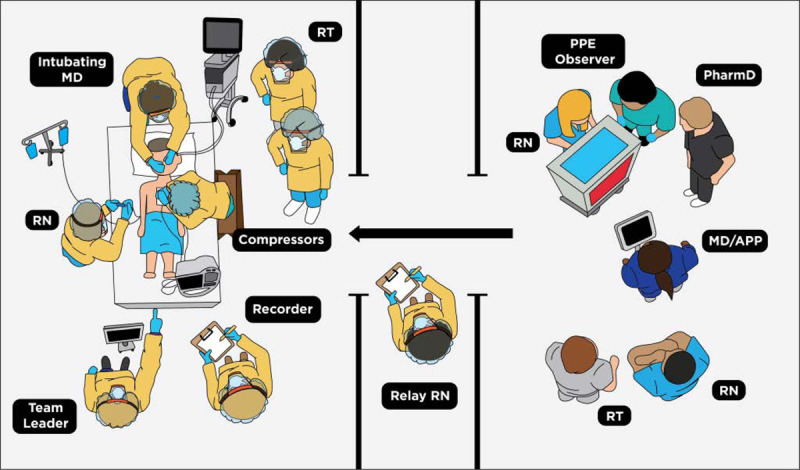
Diagram showing roles for members of the code team for a COVID-19 positive patient. The diagram shows a minimal number of direct code members inside the room in full PPE. Outside of the room, there is a group of code support members to assist with equipment and medications. In the anteroom, there is a relay nurse in full PPE to help communicate needs to the outside and bring equipment and medications to the inside. Adapted with permission by Brittany Volk from the original design by Matt Nelson from Cincinnati Children’s Media Lab at the direction of Ken Tegtmeyer MD. APP, advanced practice provider; MD, medical doctor; RN, registered nurse; RT, respiratory therapist.

Next, the simulation team performed an in situ simulation to test the guideline while conforming to institutional and governmental social distancing recommendations. Ultimately, the simulation scenario required 6 participants for each component and approximately 4 hours to perform. We broke the simulation into segments that could be filmed separately, including the bedside team for intubation, the bedside team for CPR, the relay nurse interacting with the bedside team during CPR, and the team outside of the room interacting with the relay nurse during CPR. The separation of these simulation experiences allowed for essential team members to participate in multiple roles while minimizing the total participants at 1 time to 6 to conform to social distancing recommendations. An actual resuscitation event requires a minimum of 11 HCWs during CPR based on the new guideline (Fig. 2).

Performance of these simulation events with expert-driven RCDP allowed for real-time adjustment to the guideline to achieve near-perfect practice. Simulation participants and a member of the guideline-writing committee participated in dozens of directed debriefs during time-outs. If the debrief involved guideline clarification, the simulation resumed from that stop point. If the debrief resulted in an alteration to the guideline, the simulation resumed from the beginning of that segment.

Simulation of endotracheal intubation of a COVID-19 patient with respiratory failure demonstrated the feasibility of several unique features of the guideline (Table 2). In addition to demonstrating feasibility, expert-driven RCDP during the intubation simulation scenario resulted in 3 alterations to the guideline (Table 3). First, the original guideline designated only 1 nurse at the bedside during nonemergent intubation of a COVID-19 patient to limit the exposure of HCW. Expert-driven RCDP quickly demonstrated that the bedside nurse had too many tasks, including preparing and administering induction medications, communicating with other HCW, preparing additional medications, assessing the patient, monitoring the vital signs, and charting. A participating nurse expert helped the team identify the need for a second nurse to assist with documentation, communication with other HCW during unexpected difficulties, and preparing additional medications. Second, the original guideline called for clamping of the endotracheal tube immediately after tracheal intubation followed by connecting the flow-inflating bag to the endotracheal tube with the removal of the clamp. The clamp would then be replaced when disconnecting the bag to connect the ventilator circuit. The simulation demonstrated that connecting the flow-inflating bag with a high-efficiency particulate air (HEPA) filter immediately after tracheal intubation was more efficient and provided equal protection to HCW compared to immediately clamping the endotracheal tube. Third, simulation highlighted the increased need for direct, closed-loop communication due to difficulty hearing while utilizing N95 masks.

**Table 2.** Simulation Demonstrated Feasibility of These Unique Features of the Resuscitation Guideline for COVID-19 and PUI Patients during Intubation and Cardiopulmonary Resuscitation

Endotracheal Intubation	Cardiopulmonary Resuscitation
Clear plastic Curaplex drape over the patient to minimize droplet spread	Clear plastic Curaplex drape over the patient to minimize droplet spread
Intubation by the most skilled airway specialist available	Six participants in the room during resuscitation
Rapid sequence induction	Relay nurse in the anteroom donned in full PPE to interact with the code team and the outside team
Continuous positive airway pressure preoxygenation without bagging using the two-handed mask technique	Additional team members outside of the room to assist with bringing medications and equipment to the relay nurse and placing orders in the electronic record
Video-assisted laryngoscopy rather than direct laryngoscopy	PPE observer to monitor appropriate donning and doffing of PPE when entering and exiting the room
HEPA filter on the flow-inflating bag to minimize aerosol spread	
Inflation of the endotracheal tube cuff immediately after intubation with clamping of the endotracheal tube before switching to the ventilator circuit	

**Table 3.** Guideline Alterations based on High-fidelity Simulation

	Guideline before High-fidelity Simulation	Changes Implemented after High-fidelity Simulation
Intubation	∘ Only 1 nurse in the room	∘ Two nurses in the room
	∘ Clamp endotracheal tube immediately after intubation	∘ Connect bag with HEPA filter to endotracheal tube immediately following intubation
	∘ Remove clamp after bag is attached	∘ Clamp endotracheal tube when switching from bag ventilation to the ventilator circuit
	∘ Clamp endotracheal tube again when switching to ventilator circuit	
		∘ Emphasize closed-loop communication due to difficulty hearing with N95 masks
Cardiopulmonary resuscitation	∘ Code medications obtained from code cart	∘ Consider having predrawn code medications at the bedside
	∘ Inner door of anteroom closed unless relay RN is interacting with code team	∘ Inner door of anteroom kept open for the entirety of the resuscitation event

RN, registered nurse.

The CPR simulation demonstrated the ability to perform high-quality resuscitation per the guideline with as few as 6 participants in the room. This change required the code leader to assume additional roles typically assumed by other team members, including: (1) calling for help while the nurse starts chest compressions and (2) bringing the predrawn code medications to the bedside for administration. Additionally, simulation demonstrated the feasibility of the guideline’s unique features, as shown in Table 2.

The CPR simulation also revealed several opportunities for improvement of the guideline. First, after recognition of pulselessness, there were significant delays up to 5 minutes in getting code medications due to the need to have medications outside of the patient room, don full PPE before entering the room, and maintain negative pressure inside the room. The delay in obtaining code medications demonstrated that providers should consider having code medications prepared at the bedside of a COVID-19 patient, which allows for administration within the first minute of pulselessness. Second, the closed anteroom door caused delays in the relay nurse obtaining necessary medications and equipment for the code team. This delay led to changing the guideline to open the inner anteroom door while keeping the outer anteroom door closed. During the simulation, the negative pressure monitors demonstrated adequate negative pressure with the inner anteroom door opened. The relay nurse was able to interact with the teams inside and outside of the room more freely.

## DISCUSSION

Due to the complexity of COVID-19 disease management and changes from our standard of care, we created a high-fidelity in situ simulation to test the feasibility and practice the proposed guidelines for endotracheal intubation and CPR of a COVID-19 patient. HCWs are in a unique situation during the COVID-19 pandemic, struggling as rescuers to balance the patients’ immediate needs with HCW safety. According to the CDC, as of April 9, 2020, 3% of COVID-19 cases were HCW, which is likely an underestimation because HCW status was available for only 16% of reported cases nationwide.^19^ Although critically ill pediatric cases remain rare, staffing, isolation requirements, and resource allocation should be thoughtfully considered for preparation. The clinical situations with the highest risk for HCW infection and the most significant resource utilization involving a COVID-19 patient are intubation and cardiopulmonary arrest.^6^ The need for intubation and resuscitation of a COVID-19 pediatric patient is likely to be rare, making high-fidelity in situ simulation an ideal method to prepare for this high-risk, low-frequency scenario.

Goal-directed simulation is a useful medical education method for the rapid acquisition of new skills. RCDP is a recently described simulation debriefing method that enhances learning to achieve near-perfect goal-directed practice.^20^ We used expert-driven RCDP, a novel modification to standard RCDP debriefing where all participants are considered experts rather than just the educator. Therefore, any participant can stop the scenario and debrief with the group to clarify or adjust the guideline. This unique way of debriefing allowed our team to adapt the guideline in real-time, and test guideline iterations using simulation. Also, the participation of the guideline-writing committee members in the simulation allowed for clarification of the guideline’s intent and changes to be implemented. We utilized a high-fidelity mannequin to enhance the psychological fidelity and help perfect the guideline.

Additionally, video recording of the event allowed for efficient dissemination to over 300 critical care staff as required education via a well-established online educational platform while conforming to social distancing recommendations. Video recording had further benefits because it allowed for a visual display of deviations from normal resuscitation practices that are difficult to write, such as the code team’s interaction with the relay nurse and what the patient looks like when covered with a plastic drape. Video recording also served to emphasize the need for closed-loop communication as participants were difficult to hear with full PPE.

During the pandemic, time constraints, the need for social distancing, and unnecessary use of valuable PPE for education purposes prevent extensive simulation-based education. Traditionally, developing guidelines is an iterative process that may take several days to weeks. Our simulation team tested and modified the guideline over a single 4-hour simulation event. The original intent was to spend no more than 3 hours participating in this simulation experience to minimize the amount of participants’ exposure to each other and to minimize the amount of time using the negative pressure room, a valuable resource during the COVID-19 pandemic. The additional hour required for the simulation was necessary to perfect the guideline. Significantly, no patient care was delayed or affected by the simulation.

Moreover, the participants donned full PPE to enhance the simulation’s environmental fidelity and reinforce proper PPE use for viewers of the video. Only participants who had access to repurposed N95 masks and reusable goggles participated in the simulation. The simulation group recommends this conservation practice for all institutions using high-fidelity simulation to prepare for COVID-19 patients.

Simulation helped identify several important barriers to adequate resuscitation of a COVID-19 patient. Teams managing COVID-19 patients should recognize that donning PPE will cause delays in all aspects of patient care, including the time to epinephrine during CPR. Although predrawn medications require regular disposal and result in increased costs to the patient, using predrawn medications reduced the time to epinephrine in the simulation. Reducing time to epinephrine is vital as it is associated with a significantly increased likelihood of achieving the return of spontaneous circulation.^21^ Second, using the Curaplex plastic drape is a feasible way to reduce the spread of infectious droplets and bodily fluids. However, the drape should never replace appropriate PPE as outlined by CDC guidelines for COVID-19 patients’ care during aerosol-generating procedures. To date, our critical care staff have intubated 2 COVID-19 patients using the clear plastic drape, and there has been no spread of the virus from the patient to HCW. Finally, communication will be impaired due to PPE constraints, making closed-loop communication essential.

There are several limitations to this study using expert-driven RCDP to perfect COVID-19 intubation and resuscitation guidelines. First, our institution has a process for new simulation scenarios that require review and approval from the CHST simulation center. The CHST simulation center members worked from home during the COVID-19 pandemic, so 2 trained simulation faculty members created and ran the simulation. This modification may have created bias as the 2 faculty creators also participated in the simulation. Second, a member of the guideline-writing committee was present during the simulation. Although we found this participant to be necessary to clarify the guideline’s intent during several debriefs, their presence may have decreased simulation members’ willingness to speak up and contradict the guideline. Simulation participants must be encouraged that they are experts, and their opinion is needed for the rapid acquisition of expert consensus. Finally, the need to perform this simulation in such a short time frame is a significant weakness. Although the simulation team was able to identify changes and modify the guideline, using other team members, repeating the simulation could have exposed other necessary changes.

The safety of patients and HCW is paramount during our current global crisis. As the COVID-19 pandemic is dynamic and unpredictable, HCW need to maintain proactive and resilient systems to manage the needs of patients. Simulation in situ is an efficient and effective way to test and implement significant alterations to standard guidelines. The expert-driven RCDP method demonstrated here will assist other ICUs in developing and implementing guidelines for the care of patients with COVID-19.

## DISCLOSURE

The authors have no financial interest to declare in relation to the content of this article.

## ACKNOWLEDGMENTS

We thank those individuals who participated in the video recording of this simulated event and in assistance with the finalization of the guideline including Sravya Ennamuri MD (UTSW), Renee Potera MD (UTSW), Ashley Sepanski RT (CHST), Heather Glover RN (CHST), and Carmen Yearego RT (CHST). We also acknowledge Josh Wolovits MD (UTSW) for his contribution to writing the guideline. We thank Brittany Volk for her assistance in adapting the Code Team diagram from Cincinnati Children’s Media Lab for use in our institution. We thank Matt Nelson (Medical Artist) and Ken Tegtmeyer MD (Director) from the Critical Care Medialab, Cincinnati Children’s Hospital Medical Center, for their permission to adapt their diagram of the Code Team for use in this publication.
